# First-Principle Investigations on the Electronic and Transport Properties of PbBi_2_Te_2_X_2_ (X = S/Se/Te) Monolayers

**DOI:** 10.3390/nano11112979

**Published:** 2021-11-05

**Authors:** Weiliang Ma, Jing Tian, Pascal Boulet, Marie-Christine Record

**Affiliations:** 1 IM2NP, CNRS, Faculty of Sciences, Aix-Marseille University, 13013 Marseille, France; weiliang.ma@etu.univ-amu.fr (W.M.); Jing.tian@etu.univ-amu.fr (J.T.); 2 MADIREL, CNRS, Faculty of Sciences, Aix-Marseille University, 13013 Marseille, France; pascal.boulet@univ-amu.fr

**Keywords:** 2D materials, chalcogenides, thermoelectric properties, strain effects, DFT

## Abstract

This paper reports first-principles calculations on PbBi${}_{2}$Te${}_{2}$S${}_{2}$, PbBi${}_{2}$Te${}_{2}$Se${}_{2}$ and PbBi${}_{2}$Te${}_{4}$ monolayers. The strain effects on their electronic and thermoelectric properties as well as on their stability have been investigated. Without strain, the PbBi${}_{2}$Te${}_{4}$ monolayer exhibits highest Seebeck coefficient with a maximum value of 671 μV/K. Under tensile strain the highest power factor are $12.38\times 10^{11}$ Wm${}^{-1}$K${}^{-2}$s${}^{-1}$, $10.74\times 10^{11}$ Wm${}^{-1}$K${}^{-2}$s${}^{-1}$ and $6.51\times 10^{11}$ Wm${}^{-1}$K${}^{-2}$s${}^{-1}$ for PbBi${}_{2}$Te${}_{2}$S${}_{2}$, PbBi${}_{2}$Te${}_{2}$Se${}_{2}$ and PbBi${}_{2}$Te${}_{4}$ at 3%, 2% and 1% tensile strains, respectively. These values are 85.9%, 55.0% and 3.3% larger than those of the unstrained structures.

## 1. Introduction

Thermoelectric (TE) materials that enable direct electrothermal energy conversion can have important applications in power generation [1,2], the recovery of waste heat, and on-chip cooling [3,4] and can thus provide a new route for green, clean energy to tackle the global energy crisis. However the application of TE devices has been limited by the low efficiency of their constitutive materials [5,6]. The energy conversion efficiency of TE materials is determined by the figure of merit $zT=S^{2}\sigma T/\left(\kappa_{e}+\kappa_{l}\right)$, where *S* is the Seebeck coefficient, σ is the electrical conductivity, $\kappa_{e}$ and $\kappa_{l}$ are the electronic and lattice thermal conductivities, respectively, and *T* is the temperature. As a consequence, an improvement of the TE performance requires increasing the power factor ($PF=S^{2}\sigma$) and/or reducing the total thermal conductivity. Several effective strategies such as the optimization of the carriers density, the convergence of the electronic bands [7,8], and the introduction of resonant states [9,10] have been proposed to enhance PF. For instance, Diznab [8] recently boosted the PF of Bi${}_{2}$Te${}_{3}$ monolayer by 43.6% via valence band convergence obtained through Se substitution for Te. Besides, the existence of a resonant level in Tl-doped PbTe and in Tl${}_{0.02}$Pb${}_{0.98}$TeSi${}_{0.02}$Na${}_{0.02}$ boosts the Seebeck coefficient, allowing zT for reaching a value of 1.5 [10] and 1.7 [11], respectively. Apart from band engineering, zT can also be improved by the so-called phonon engineering through reducing the material’s dimensionality or generating superlattices. This strategy has proved efficient in n-type Bi${}_{2}$Te${}_{2.7}$Se${}_{0.3}$ nanowires with a 13% zT improvement [12].

Among many TE materials systems proposed in the past decades, complex layered chalcogenides are potential candidates for TE applications due to their low $\kappa_{l}$. Based on the methods mentioned above, the zT value has been pushed up to 2.2 for phase-separated PbTe${}_{0.7}$S${}_{0.3}$ [13], 1.86 for Bi${}_{0.5}$Sb${}_{1.5}$Te${}_{3}$ [14] and 2.5 for PbTe-8%SrTe [15]. Furthermore, experimental measurements and theoretical calculations reveal that monolayer structures are promising for future TE applications [12,16,17], since they benefit from the combination of two complementary approaches, namely the electronic band engineering and the phonon one. As reported in literature for MoS${}_{2}$, Bi${}_{2}$Te${}_{3}$ and Bi${}_{2}$Se${}_{3}$ [18,19,20], monolayer or few-layer nanosheets can be experimentally obtained by exfoliation from the bulk, or synthesized by solution-phase method as with PbBi${}_{2}$Te${}_{4}$ and Pb${}_{2}$Bi${}_{2}$Te${}_{5}$ [21]. Due to their layered structures involving van der Waal interactions, these latter compounds present additional interest for future TE application, namely an intrinsically low thermal conductivity and the possibility to obtain few-layer thick nanosheets by exfoliation from the bulk.

The bulk compounds in the n(PbTe)-m(Bi${}_{2}$Te${}_{3}$) system bearing a layered structure are the following: Bi${}_{2}$Te${}_{3}$ with a quintuple layers structure and sequence -Te-Bi-Te-Bi-Te-, PbBi${}_{2}$Te${}_{4}$ with a septenary layers structure and sequence -Te-Bi-Te-Pb-Te-Bi-Te-, and Pb${}_{2}$Bi${}_{2}$Te${}_{5}$ with an ennead layers structure and sequence -Te-Bi-Te-Pb-Te-Pb-Te-Bi-Te-. Among them, topologically protected surface states have been found in Bi${}_{2}$Te${}_{3}$ thin film [22], PbBi${}_{2}$Te${}_{2}$Se${}_{2}$ monolayer [23] and PbBi${}_{2}$Te${}_{4}$ bilayer [24], leading to the intrinsic convergence of multivalley bands, which is the most interesting for improving TE properties.Benefiting from band convergence and quantum confinement, the single quintuple tetradymites family of Bi${}_{2}$X${}_{3}$ (X = S, Se, Te) exhibits high *zT* values of 1.4–2.4 [8,25,26] and have been widely investigated to date. Hence, in this study, we have focused our investigation on the PbBi${}_{2}$Te${}_{4}$ nanosheet and PbBi${}_{2}$Te${}_{2}$Se${}_{2}$ and PbBi${}_{2}$Te${}_{2}$S${}_{2}$ ones, which have been obtained by substituting Se and S for two Te atoms in PbBi${}_{2}$Te${}_{4}$. Using DFT calculations, we have determined the stability, the electronic structure, the TE properties, and the thermal conductivity of these nanosheets. We have also explored the effect of bi-axial strains on their properties.

## 2. Materials and Methods

DFT calculations have been performed using the all-electron FP-LAPW approach with local orbital method as implemented in WIEN2K [27]. To obtain a good convergence, the plane wave cut-off criterium $R_{mt}K_{max}$ was set to 9.0, and the k-meshes used to sample the Brillouin zone have been set to 12×12×1 for structural optimization and 16×16×1 for self-consistent energy calculations. The total energy and atomic forces convergence thresholds have been defined as 0.068 meV and 0.257 meV/Å for the three compounds PbBi${}_{2}$Te${}_{2}$S${}_{2}$, PbBi${}_{2}$Te${}_{2}$Se${}_{2}$ and PbBi${}_{2}$Te${}_{4}$. The energy separation between the core and valence electrons has been fixed at −5.0 Ry. The electronic transport properties, namely *S*, $\kappa_{e}$, and σ have been calculated by solving the Boltzmann semi-classical transport equation as implemented in BoltzTraP2 [28]. The implementation of BoltzTraP2 is based on the use of full band structure in the Brillouin zone (BZ). Herein, the BZ has been sampled using a dense k-mesh of 64×4×8, and we have checked that the interpolation of the band structure performed by BoltzTrap2 properly reproduced the DFT band structure.

Second and third order anharmonic interatomic force constants (IFCs) have been calculated by means of the DFPT method by using the QUANTUM-ESPRESSO package [29] together with the Phonopy and Phono3py programs [30]. A supercell of 5×5×1 with a k-mesh of 4×4×2 and a supercell of 4×4×1 with a Γ k-point calculation have been considered for second and third order IFCs evaluations, respectively. The calculation is carried out by using the projector augmented-wave pseudopotential method with a plane-wave energy cutoff of 70 Ry (952 eV) and a total force threshold of 10${}^{-4}$ Ry/bohr. In subsequent post-processing calculations, phonon lifetimes have been sampled using a 43×43×7 mesh. The lattice thermal conductivity has been calculated by using both a full solution of the linearized phonon Boltzmann equation (LBTE) method as introduced in ref. [31] and the relaxation time approximation (RTA) method. Within the RTA method, the lattice thermal conductivity tensor $\kappa_{l}^{\alpha \beta }$ is expressed as

$$\kappa_{l}^{\alpha \beta }=\frac{1}{NV_{0}}\sum_{\lambda }C_{\lambda }v_{\lambda }^{\alpha }v_{\lambda }^{\beta }\tau_{\lambda }\;,$$

where *N* is the number of q-points, $V_{0}$ is the unit cell volume, $v_{\lambda }$ is the group velocity indexed with the Cartesian coordinates α and β, and $\tau_{\lambda }$ is the phonon scattering time for the specific phonon mode λ. The heat capacity for the specific phonon mode with frequency $\omega_{\lambda }$ is $C_{\lambda }=k_{B}{\left(\frac{\hbar^{2}\omega_{\lambda }}{k_{B}T}\right)}^{2}n_{\lambda }^{0}\left(n_{\lambda }^{0}+1\right)$, where $n_{\lambda }^{0}$ is the Bose-Einstein distribution function. The spectral representation of the dynamical thermal conductivity obtained from the LBTE method is $\kappa_{l}=\int d\omega^{\prime }\frac{\rho (\omega^{\prime })}{\omega^{\prime }-i\omega }$, where $\rho (\omega^{\prime })$ is the spectral density. Furthermore, because the lattice thermal conductivity is an intensive property for bulk materials, that of two-dimensional material should be normalized by multiplying by $L_{z}/d$, where $L_{z}$ is the lattice parameter *c* and *d* is the thickness of the nanosheet.

## 3. Results

### 3.1. Structural Data

**Figure 1:**
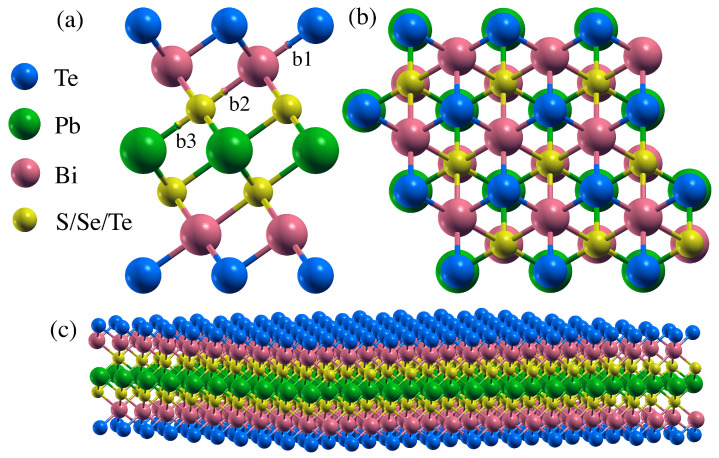
Side view (**a**), top view (**b**) and extended view (**c**) of PbBi${}_{2}$Te${}_{2}$X${}_{2}$ (X = S, Se, Te). The three non-equivalent bonds (*b*1–*b*3) are shown in (**a**).

Bulk PbBi${}_{2}$Te${}_{4}$ crystallizes in a rhombohedral lattice system (R$\bar{3}$m) with seven atoms located along the c-axis (Supplementary Materials Figure S1a). However, PbBi${}_{2}$Te${}_{4}$ can also be treated as a hexagonal cell (Figure S1b) constituted by three seven-atom-layered slabs with three non-equivalent bonds each, held together by van de Waals interactions. One of these slabs is presented in Figure 1. It clearly shows seven atomic layers (one of Pb, two of Bi, two outmost Te layers and two inner Te ones) bonded by three non-equivalent bonds. To avoid spurious interaction between neighboring layers, the PbBi${}_{2}$Te${}_{2}$S${}_{2}$, PbBi${}_{2}$Te${}_{2}$Se${}_{2}$ and PbBi${}_{2}$Te${}_{4}$ nanosheets have been optimized with an on-top vacuum thickness of 1.6 nm. Based on our previous work on bulk Bi${}_{2}$Te${}_{3}$, PbBi${}_{2}$Te${}_{4}$ and Pb${}_{2}$Bi${}_{2}$Te${}_{5}$ [32], all of the in-layer bonds are neither pure ionic bonds nor pure covalent ones, the covalent contribution being increased when the material is subjected to compressive strains. Since both the size of the gap between slabs and the inter slabs X-X distances are also increased under compressive strains, we should expect a similar trend in the nanosheet that corresponds to an isolated slab, i.e., b1 should be more ionic than b2 and b3. If one replaces the inner Te atom by a more electronegative Se or S one, the outermost Te atom gets much less electron-rich.

The equilibrium lattice constants of PbBi${}_{2}$Te${}_{2}$S${}_{2}$, PbBi${}_{2}$Te${}_{2}$Se${}_{2}$ and PbBi${}_{2}$Te${}_{4}$ have been calculated using the WC-GGA functional [33] without spin-orbit coupling (SOC) for both bulk and nanosheet structures. The results are listed in Table 1. For bulk PbBi${}_{2}$Te${}_{4}$, the optimized lattice constants are a=0.443 nm, c=4.156 nm and the slab thickness is 1.127 nm, which is in good agreement with reported experimental values [34]. As to the PbBi${}_{2}$Te${}_{4}$ nanosheet, the thickness is 1.120 nm, which is close to that of the slab in the bulk and to that reported in the literature [21]. In the septuple layers slab, each Pb atom binds with six Te atoms with identical bond length (b3=0.3210 nm), while each Bi atom binds with six Te atoms with two sets of three identical bond lengths (b1=0.3070 nm and b2=0.3248 nm). If the inner Te atoms are replaced by S or Se ones, the corresponding slab thickness and bond lengths decrease.

### 3.2. Electronic and Transport Properties

**Figure 2:**
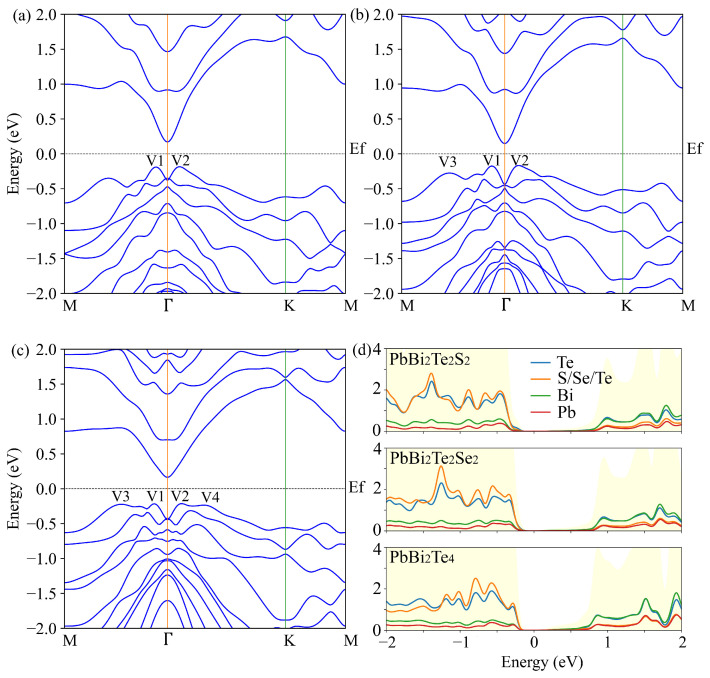
Electronic band structures of PbBi${}_{2}$Te${}_{2}$S${}_{2}$ (**a**), PbBi${}_{2}$Te${}_{2}$Se${}_{2}$ (**b**), PbBi${}_{2}$Te${}_{4}$ (**c**) and corresponding total (light yellow background) and partial (color lines) DOS (**d**) calculated with the WC-GGA functional and SOC.

As shown in Figure 2, PbBi${}_{2}$Te${}_{2}$S${}_{2}$, PbBi${}_{2}$Te${}_{2}$Se${}_{2}$ and PbBi${}_{2}$Te${}_{4}$ nanosheets are semiconductors with indirect energy band gaps of 0.354 eV, 0.314 eV and 0.376 eV, respectively. The band structure of PbBi${}_{2}$Te${}_{4}$ calculated with WC-GGA is compared in Figure S2 with that calculated with the HSE06 hybrid functional [35]. Except for the band gap, which is substantially enlarged with HSE06 (0.967 eV), both functionals qualitatively give the same results. The same observation can be done for PbBi${}_{2}$Te${}_{2}$Se${}_{2}$ and PbBi${}_{2}$Te${}_{2}$S${}_{2}$. Since the band gaps calculated with the WC-GGA functional are in better agreement with those reported in literature for nanosheets of homologous Pb${}_{m}$Bi${}_{2n}$Te${}_{3n+m}$ compounds, which all belong to the range 0.25–0.7 eV [21], and the hybrid HSE06 functional has not been found superior to pure DFT ones in the calculations of band structures and thermoelectric properties of tetradymite materials [36], we have been using the WC-GGA functional in this work. In all the three compounds, PbBi${}_{2}$Te${}_{2}$S${}_{2}$, PbBi${}_{2}$Te${}_{2}$Se${}_{2}$ and PbBi${}_{2}$Te${}_{4}$, the conduction band minimum (VBM) is located at the Γ point and the valence band maximum (VBM) is located along the Γ-*K* direction (Figure 2). In contrast to a single conduction band minimum, two, three and four valence band maxima (V1,V2,V3,V4) located within a small range of 0.1 eV wide are observed near the Fermi energy for PbBi${}_{2}$Te${}_{2}$S${}_{2}$, PbBi${}_{2}$Te${}_{2}$Se${}_{2}$ and PbBi${}_{2}$Te${}_{4}$, respectively. In contrast to observations made in Bi${}_{2}$Te${}_{3}$ monolayer [8], the substitution of Se for Te in the PbBi${}_{2}$Te${}_{4}$ monolayer does not lead to high valence band degeneracy $N_{v}$. Compared with the conduction band, the valence band is less dispersed, leading to a higher total DOS slope and thus promising higher Seebeck coefficient for p-type material.

The analysis of partial density of states (PDOS) (see Figure S3) reveals that Te-5p, S/Se/Te-5p, Bi-6s and Pb-6s orbitals dominate the valence band near the Fermi energy, while the conduction band is dominated by Bi-6p, Pb-6p and S/Se/Te-5p orbitals. A slight contribution of the Pb-6p orbital in the valence band around the Fermi level is also evidenced. It increases for the PbBi${}_{2}$Te${}_{2}$X${}_{2}$ compounds along the Te, Se and S sequence.

The bulk modulus *B*, elastic constants, effective mass and the cohesive energy of the compounds of interest have been calculated and the values are reported in Table 2. When X in PbBi${}_{2}$Te${}_{2}$X${}_{2}$ follows the sequence Te, Se, S, the bulk modulus B increases, indicating a bond strengthening, which can be associated to the electronegativity increase of the chalcogen. One can note that, in agreement with the evolution of the band structure (Figure 2), the calculated effective mass increases with the change of inner chalcogenide layer from S to Se and Te. Indeed the top valence orbitals and bottom conduction orbitals are getting softer, leading to heavier effective mass and lower carriers mobility.

The elastic constants calculations allows for characterizing the mechanical stability of the nanosheets. The necessary and sufficient conditions of mechanical stability for the rhombohedral I system are given in Ref. [37] as

$$\begin{array}{ccc} C_{11} & > & |C_{12}|;\;C_{44}>0\\ C_{13}^{2} & < & \frac{1}{2}C_{33}\left(C_{11}+C_{12}\right)\\ C_{14}^{2} & < & \frac{1}{2}C_{44}\left(C_{11}-C_{12}\right) \end{array}$$

Our calculations show that the PbBi${}_{2}$Te${}_{2}$S${}_{2}$, PbBi${}_{2}$Te${}_{2}$Se${}_{2}$ and PbBi${}_{2}$Te${}_{4}$ monolayers are mechanically stable. Furthermore, the cohesive energies have been evaluated with the general formula: $E_{coh}=E_{tot}-\sum_{i}E_{i}$, where $E_{tot}$ is the total energy of the monolayer, and $E_{i}$ is the energy of each constitutive atom. The negative values at 0 K of the cohesive energies, namely −3.22 eV/at., −3.09 eV/at. and −2.93 eV/at. for PbBi${}_{2}$Te${}_{2}$S${}_{2}$, PbBi${}_{2}$Te${}_{2}$Se${}_{2}$ and PbBi${}_{2}$Te${}_{4}$, respectively, also support the nanosheet stability.

**Figure 3:**
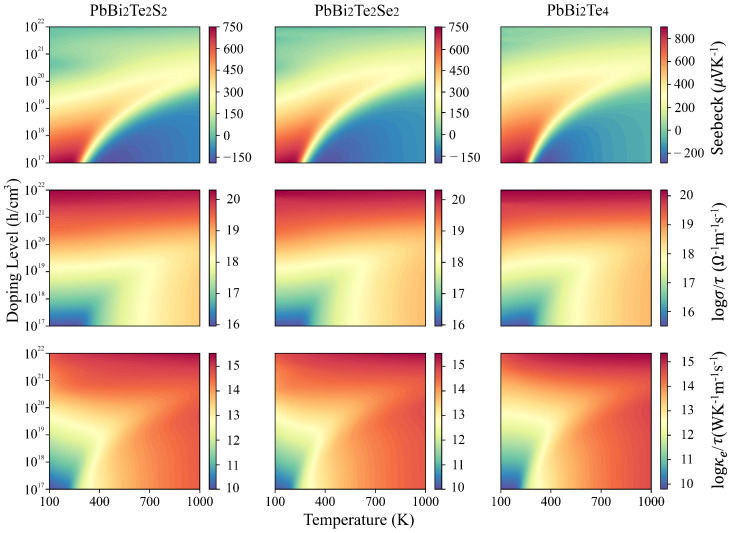
Calculated Seebeck coefficient and τ-scaled electrical and electronic thermal conductivities in the a-axis direction versus temperature and p-type doping levels for PbBi${}_{2}$Te${}_{2}$S${}_{2}$, PbBi${}_{2}$Te${}_{2}$Se${}_{2}$ and PbBi${}_{2}$Te${}_{4}$.

Based on the above considerations, we present in Figure 3 the temperature and p-type doping dependence of the thermoelectric properties (Seebeck coefficient *S*, electrical conductivity σ/τ and electronic thermal conductivity $\kappa_{e}/\tau$) in the a-axis direction. The optimum Seebeck coefficient appears for the doping levels $10^{17}$ to $5\times 10^{19}$ h/cm${}^{3}$ and the low to intermediate 100–400 K temperatures, where both σ/τ and $\kappa_{e}/\tau$ are low. The largest Seebeck coefficients at room temperature are 601 μV/K, 559 μV/K, 671 μV/K for PbBi${}_{2}$Te${}_{2}$S${}_{2}$, PbBi${}_{2}$Te${}_{2}$Se${}_{2}$ and PbBi${}_{2}$Te${}_{4}$, respectively.

### 3.3. Lattice Thermal Conductivity

**Figure 4:**
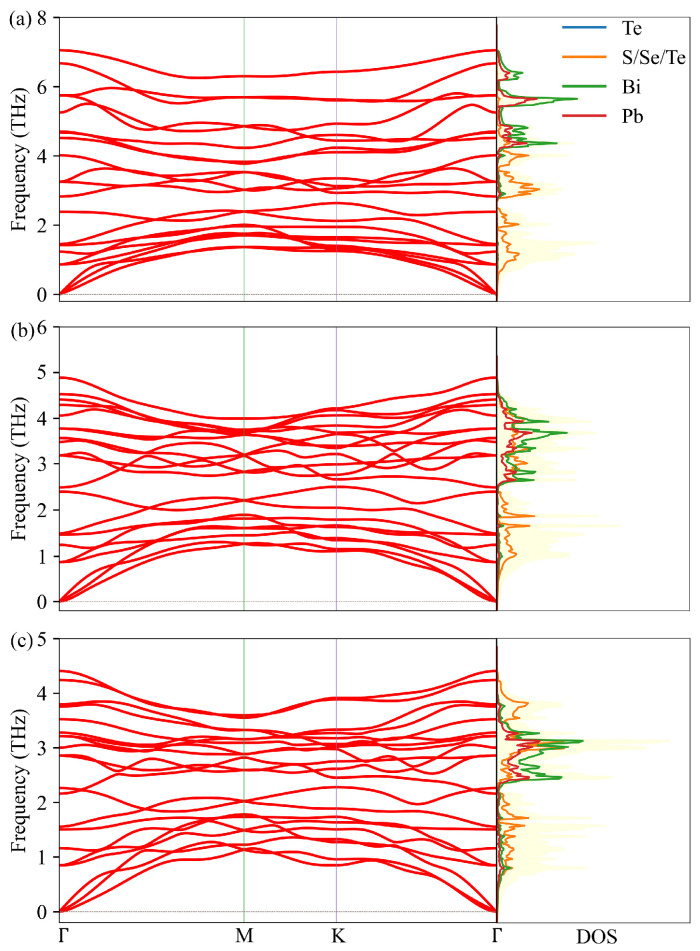
Phonon spectrum curves of PbBi${}_{2}$Te${}_{2}$S${}_{2}$ (**a**), PbBi${}_{2}$Te${}_{2}$Se${}_{2}$ (**b**), PbBi${}_{2}$Te${}_{4}$ (**c**) and corresponding total DOS (light yellow background) and projected DOS (color lines).

The harmonic phonon spectrum depends weakly on the choice of the functional [38]. In addition, it has been reported that the LDA functionals [39] consistently give a proper bulk modulus, resulting in a better agreement with experiment for Bi${}_{2}$Te${}_{3}$ [40]. Hence the LDA functionals have been chosen to determine the harmonic and anharmonic IFCs. The PbBi${}_{2}$Te${}_{2}$S${}_{2}$, PbBi${}_{2}$Te${}_{2}$Se${}_{2}$ and PbBi${}_{2}$Te${}_{4}$ monolayers have been reoptimized to their relaxed states. The obtained equilibrium lattice constants are a=0.417 nm, c=1.032 nm for PbBi${}_{2}$Te${}_{2}$S${}_{2}$, a=0.424 nm, c=1.065 nm for PbBi${}_{2}$Te${}_{2}$Se${}_{2}$ and a=0.436 nm, c=1.118 nm for PbBi${}_{2}$Te${}_{4}$, which are quite close to the lattice constants obtained in Section 3.1. The phonon dispersion curves together with the DOS of PbBi${}_{2}$Te${}_{2}$S${}_{2}$, PbBi${}_{2}$Te${}_{2}$Se${}_{2}$ and PbBi${}_{2}$Te${}_{4}$ are shown in Figure 4 depicting 3 acoustic and 18 optical branches. The longitudinal optical (LO)-transverse optical (TO) splitting at the Γ point is particularly large on the phonon dispersion in the m(PbTe)-n(Bi${}_{2}$Te${}_{3}$) system compounds [41,42], which is caused by large Born effective charges. Therefore, the contribution of the non-analytical term to the dynamical matrix has been considered and the calculated Born effective charges by the Berry phase method [43] and dielectric constants are shown in Table S1. All the monolayer crystals are dynamically stable with no imaginary modes through the whole BZ. To further acertain the thermodynamic stability of the compounds, the Gibbs energy *G* has been calculated by taking into account the vibrational part of the partition function. The procedure is described in the supplemental data. Negative *G* values have been found for the investigated monolayers in the temperature range 0–1000 K (see Figures S4 and S5), suggesting that they are all stable. PbBi${}_{2}$Te${}_{2}$S${}_{2}$ and PbBi${}_{2}$Te${}_{2}$Se${}_{2}$ have similar dispersion curves with strongly interlaced optical and acoustic modes and small frequency gaps at 2.4 THz for PbBi${}_{2}$Te${}_{2}$Se${}_{2}$ and 2.7 THz for PbBi${}_{2}$Te${}_{2}$S${}_{2}$. For PbBi${}_{2}$Te${}_{4}$, there is less crossing between optical and acoustic branches, which will play an important role in the acoustic + acoustic → optical scattering. Furthermore, the maximum frequencies of the acoustic phonon modes are 1.69 THz, 1.51 THz and 1.38 THz, and that of the optical phonon modes are 7.05 THz, 4.89 THz and 4.41 THz for PbBi${}_{2}$Te${}_{2}$Se${}_{2}$, PbBi${}_{2}$Te${}_{2}$S${}_{2}$, and PbBi${}_{2}$Te${}_{4}$, respectively.

**Figure 5:**
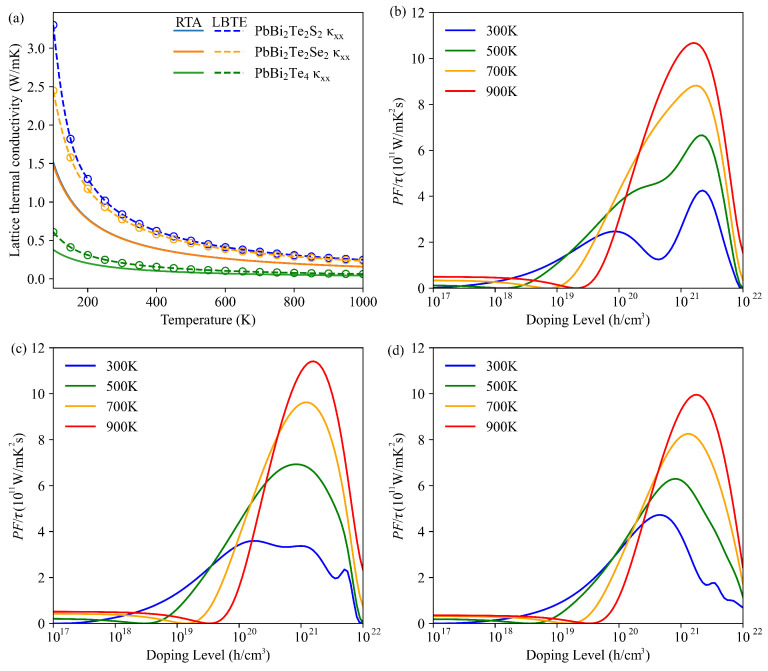
(**a**) Lattice thermal conductivity of the PbBi${}_{2}$Te${}_{2}$S${}_{2}$, PbBi${}_{2}$Te${}_{2}$Se${}_{2}$ and PbBi${}_{2}$Te${}_{4}$ as a function of temperature obtained from LBTE (dash lines) and RTA (solid lines) in the a-axis direction. Figure of merit versus hole doping level at various temperatures for PbBi${}_{2}$Te${}_{2}$S${}_{2}$ (**b**), PbBi${}_{2}$Te${}_{2}$Se${}_{2}$ (**c**), and PbBi${}_{2}$Te${}_{4}$ (**d**).

The lattice thermal conductivity $\kappa_{l}$ evaluated by solving the Boltzmann transport equation (BTE) with LBTE and RTA methods is shown in Figure 5a. Contrary to RTA, the LBTE gives a rigorous way to evaluate lattice thermal conductivity by considering phonon–phonon interactions, but it necessitates huge calculations. From the LBTE method, the $\kappa_{l}$ at room temperature of PbBi${}_{2}$Te${}_{2}$S${}_{2}$, PbBi${}_{2}$Te${}_{2}$Se${}_{2}$ and PbBi${}_{2}$Te${}_{4}$ are 0.84 Wm${}^{-1}$K${}^{-1}$, 0.79 Wm${}^{-1}$K${}^{-1}$ and 0.21 Wm${}^{-1}$K${}^{-1}$, respectively. Although $\kappa_{l}$ is underestimated by RTA, it is still a useful method to evaluate the phonon transport through the phonon mode group velocities and lifetimes calculations (see Figure S6). Both the average phonon lifetime and average phonon group velocity of PbBi${}_{2}$Te${}_{4}$ (0.64 ps and 0.32 km s${}^{-1}$) are substantially lower than those of PbBi${}_{2}$Te${}_{2}$Se${}_{2}$ (1.75 ps and 0.36 km s${}^{-1}$) and PbBi${}_{2}$Te${}_{2}$S${}_{2}$ (1.51 ps and 0.39 km s${}^{-1}$). The detailed analysis shows that the contribution of the acoustic modes to the velocity is approximately the same in the three structures. It is noticeable that, in PbBi${}_{2}$Te${}_{2}$S${}_{2}$, optical modes above 5 THz are particularly prominent with high velocity whereas they are absent in PbBi${}_{2}$Te${}_{2}$Se${}_{2}$ and PbBi${}_{2}$Te${}_{4}$. In addition, irrespective of the frequency domain, the phonon life time is larger for PbBi${}_{2}$Te${}_{2}$Se${}_{2}$ and PbBi${}_{2}$Te${}_{2}$S${}_{2}$ than for PbBi${}_{2}$Te${}_{4}$. Theses observations explain why $\kappa_{l}$ of PbBi${}_{2}$Te${}_{4}$ is lower than that of PbBi${}_{2}$Te${}_{2}$S${}_{2}$ to PbBi${}_{2}$Te${}_{2}$Se${}_{2}$. Slack [44] reported that intrinsically high lattice thermal conductivity can be obtained by low average atomic mass, strong interatomic bonding, simple crystal structure and strong anharmonic interaction. Since PbBi${}_{2}$Te${}_{2}$S${}_{2}$, PbBi${}_{2}$Te${}_{2}$Se${}_{2}$ and PbBi${}_{2}$Te${}_{4}$ bear roughly opposite characteristics to those just exposed, they are expected to have low lattice thermal conductivity. As mentioned above, more electronegative atoms such as Se or S compared to Te, are expected to share stronger bonding with neighbors (Pb and Bi), leading to higher lattice thermal conductivity. The trend observed in the bulk modulus of PbBi${}_{2}$Te${}_{2}$S${}_{2}$ (30.33 GPa), PbBi${}_{2}$Te${}_{2}$Se${}_{2}$ (28.75 GPa) and PbBi${}_{2}$Te${}_{4}$ (26.05 GPa) also support the bonding strength trend and the lattice thermal conductivity one.

The τ-scaled power factor is depicted in Figure 5b,c for PbBi${}_{2}$Te${}_{2}$S${}_{2}$, PbBi${}_{2}$Te${}_{2}$Se${}_{2}$ and PbBi${}_{2}$Te${}_{4}$ as a function of the whole doping level and at temperatures 300 K, 500 K, 700 K and 900 K. The observed tendency is the same for all the compounds, namely, the maximum peak of the power factor increases with temperature, except for 300 K, where it is noticeable that the power factor of PbBi${}_{2}$Te${}_{2}$S${}_{2}$ and PbBi${}_{2}$Te${}_{2}$Se${}_{2}$ are about the same, whereas that of PbBi${}_{2}$Te${}_{4}$ is obviously lower.

### 3.4. Strain Engineering of Electronic and Phonon Transport Properties

In this section we investigate the effects of in-plane biaxial strains on the electronic and phonon transport properties of the PbBi${}_{2}$Te${}_{2}$S${}_{2}$, PbBi${}_{2}$Te${}_{2}$Se${}_{2}$ and PbBi${}_{2}$Te${}_{4}$ monolayers. The positive (negative) values of strain η, which indicates the magnitude of relative tensile (compressive) strain along the a and b directions, have been calculated as $\eta =\left(a-a_{0}\right)/a_{0}$. In this work, the in-plane strains vary from −3% to 3% and the cross-plane c lattice parameter and atomic positions for each η have been optimized until the total energy and atomic forces reached their minimum. The optimized lattice parameters and total energy of the structures are listed in Table 3. The lattice constant c of the relaxed structure decreases approximately linearly under the in-plane strain changing from −3% to 3%, with a slope of 0.012 nm per unit percentage (1.15% of the lattice constant c), which shows strong coupling between a and c.

**Figure 6:**
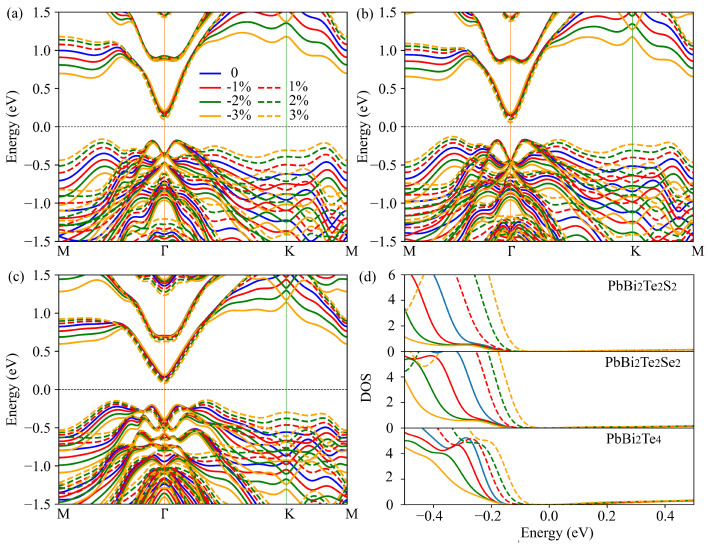
Electronic bands structure of PbBi${}_{2}$Te${}_{2}$S${}_{2}$ (**a**), PbBi${}_{2}$Te${}_{2}$Se${}_{2}$ (**b**) and PbBi${}_{2}$Te${}_{4}$ (**c**) under applied strain and their corresponding total DOS (**d**).

To evidence the strain effect on the electronic structure, the bands structures and DOS of the strained and unstrained PbBi${}_{2}$Te${}_{2}$S${}_{2}$, PbBi${}_{2}$Te${}_{2}$Se${}_{2}$ and PbBi${}_{2}$Te${}_{4}$ monolayers are shown in Figure 6. The energy gap decreases slightly as the applied strain (from −3% to 3%) increases, although not leading to a semiconductor-metal transition. More interestingly, as shown in Figure 6, the valence band around Γ is very robust under strains whereas secondary valence band maxima rise in energy with the increasing tensile strains, which provides an opportunity to boost the thermoelectric properties via valence bands degeneracy. By applying strains, the derivative of the valence bands total DOS first increases and then decreases, especially for PbBi${}_{2}$Te${}_{4}$. Following the band theory, the hole contribution to the Seebeck coefficient is given as [45]: $S=\frac{k_{B}}{e}\left[2+\ln\left(\frac{N_{V}}{p}\right)\right]$, where $N_{V}$ and *p* are the effective DOS and the number of hole carriers, respectively. Therefore, a slight tensile strain should lead to a higher Seebeck coefficient. This result is in agreement with previously reported ones. Indeed, it has been shown that slight tensile strains applied on p-type Pb${}_{2}$Bi${}_{2}$Te${}_{5}$ increase the PF [32].

**Figure 7:**
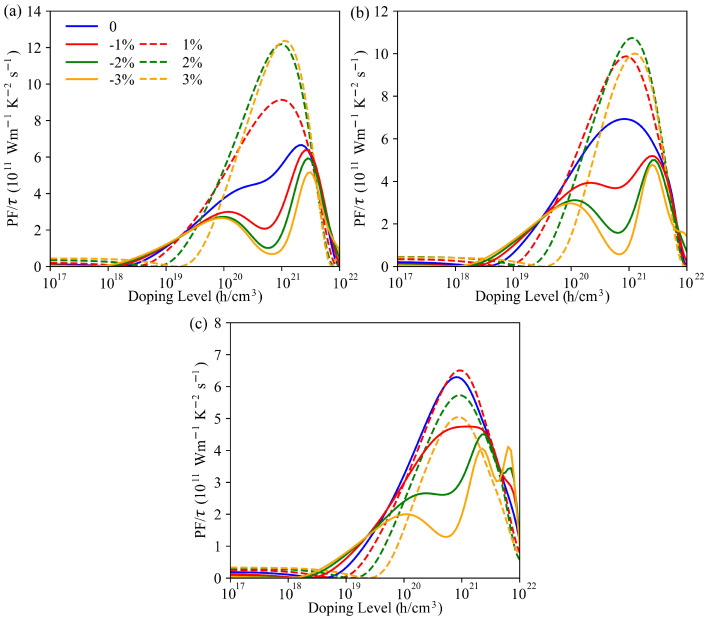
τ-scaled power factor of PbBi${}_{2}$Te${}_{2}$S${}_{2}$ (**a**), PbBi${}_{2}$Te${}_{2}$Se${}_{2}$ (**b**) and PbBi${}_{2}$Te${}_{4}$ (**c**) monolayers versus p-type doping level for various compressive and tensile strains in a-axis direction at 500 K.

The τ-scaled PF of the PbBi${}_{2}$Te${}_{2}$S${}_{2}$, PbBi${}_{2}$Te${}_{2}$Se${}_{2}$ and PbBi${}_{2}$Te${}_{4}$ monolayers at 500 K as a function of carrier concentration are plotted in Figure 7. In all cases, the maximum PF with p-type doping increases first and then decreases with increasing applied tensile strains. This behavior can be seen for PbBi${}_{2}$Te${}_{2}$S${}_{2}$ in Figure S7, which shows the PF evolution up to 4% tensile strain.

The maximum PF values are found to be $12.30\times 10^{11}$ Wm${}^{-1}$K${}^{-2}$s${}^{-1}$, $10.74\times 10^{11}$ Wm${}^{-1}$K${}^{-2}$s${}^{-1}$ and $6.51\times 10^{11}$ Wm${}^{-1}$K${}^{-2}$s${}^{-1}$ for PbBi${}_{2}$Te${}_{2}$S${}_{2}$, PbBi${}_{2}$Te${}_{2}$Se${}_{2}$ and PbBi${}_{2}$Te${}_{4}$ at 3%, 2% and 1% tensile strains, respectively; they are 85.9%, 55.0% and 3.3% larger than those of unstrained structures. Therefore, it appears that an appropriate mean of optimizing the thermoelectric properties of PbBi${}_{2}$Te${}_{4}$ nanosheet is to substitute S/Se for Te in the inner layers and subject it to a tensile strain.

**Figure 8:**
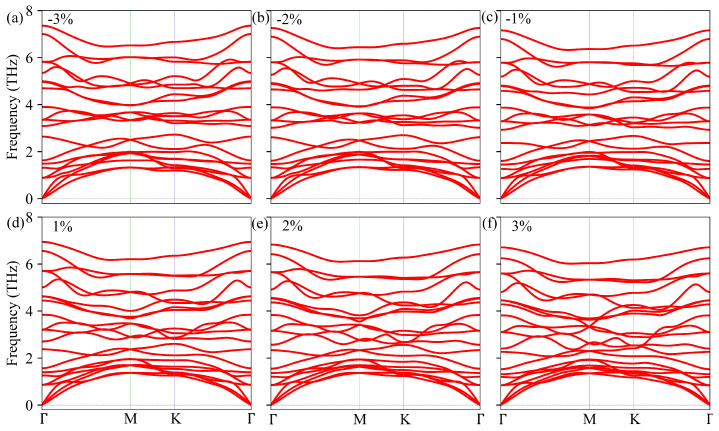
Phonon spectrum curves of Pb Bi${}_{2}$Te${}_{2}$S${}_{2}$ monolayer under −3% (**a**), −2% (**b**) and −1% (**c**) compressive strains, and 1% (**d**), 2% (**e**) and 3% (**f**) tensile strains.

Using the same scheme as for unstrained structure, the anharmonic force constants as well as the Born effective charges and dielectric constants under strains have been calculated. The phonon spectrum curves of PbBi${}_{2}$Te${}_{2}$S${}_{2}$, PbBi${}_{2}$Te${}_{2}$Se${}_{2}$ and PbBi${}_{2}$Te${}_{4}$ along high symmetry directions are plotted in Figure 8, Figures S8 and S9, respectively. Irrespective of the strain, no imaginary phonon modes are found in the phonon spectrum of PbBi${}_{2}$Te${}_{2}$S${}_{2}$ and PbBi${}_{2}$Te${}_{2}$Se${}_{2}$. By contrast, PbBi${}_{2}$Te${}_{4}$ shows imaginary phonon modes under −3%, −2% and 3% strains.

When strain goes from −3% to +3%, the maximum frequency of the optical and acoustic phonon modes for PbBi${}_{2}$Te${}_{2}$S${}_{2}$ decrease from 7.36 Thz to 6.71 Thz, and from 1.63 THz to 1.57 THz, respectively. According to the Slack equation [44], there is a negative correlation between $\kappa_{l}$ and the Debye temperature, which can be defined as $\theta_{i}=\frac{\hbar \omega_{i}}{k_{B}}$ [46], where $\omega_{i}$ is the frequency of phonon mode boundary. When the strain varies from −3% to 3%, the decrease of the maximum frequency indicates a decrease of the Debye temperature, leading to more activated phonon modes, higher phonon scattering rates and hence lower lattice thermal conductivity.

## 4. Conclusions

In summary, we have performed first-principle calculations of the electronic structure, the thermoelectric properties, the stability and the strain-engineering effects on PbBi${}_{2}$Te${}_{2}$S${}_{2}$, PbBi${}_{2}$Te${}_{2}$Se${}_{2}$ and PbBi${}_{2}$Te${}_{4}$ monolayers. All the three monolayers of interest are narrow-gap semiconductors with an indirect band gap and are energetically and thermodynamically stable without strain. In these conditions, compared with PbBi${}_{2}$Te${}_{2}$S${}_{2}$ and PbBi${}_{2}$Te${}_{2}$Se${}_{2}$, PbBi${}_{2}$Te${}_{4}$ presents a higher Seebeck coefficient, lower electrical conductivity and lower electronic thermal conductivity. The maximum Seebeck coefficient of PbBi${}_{2}$Te${}_{4}$ monolayer is 671 μV/K. Under small strains, the bands structures near Γ are very robust, whereas secondary valence band maxima rise in energy, leading to a valence bands alignment near the Fermi level. The highest PF/τ values are $12.38\times 10^{11}$ Wm${}^{-1}$K${}^{-2}$s${}^{-1}$, $10.74\times 10^{11}$ Wm${}^{-1}$K${}^{-2}$s${}^{-1}$ and $6.51\times 10^{11}$ Wm${}^{-1}$K${}^{-2}$s${}^{-1}$ for PbBi${}_{2}$Te${}_{2}$S${}_{2}$, PbBi${}_{2}$Te${}_{2}$Se${}_{2}$ and PbBi${}_{2}$Te${}_{4}$ at 3%, 2% and 1% tensile strains respectively. These values, which are 85.9%, 55.0% and 3.3% larger than those of the unstrained structures, prove that strain engineering is an effective approach to enhance thermoelectric properties.

## Figures and Tables

**Table 1:** Calculated lattice constants, slab thickness and bond lengths as labeled in Figure 1 (b1 is Te-Bi, b2 is Bi-S/Se/Te and b3 is S/Se/Te-Pb) of PbBi${}_{2}$Te${}_{2}$X${}_{2}$ (X = S, Se, Te) in bulk and nanosheet structures optimized with the WC functional. All data in nm.

		PbBi${}_{2}$Te${}_{2}$S${}_{2}$	PbBi${}_{2}$Te${}_{2}$Se${}_{2}$	PbBi${}_{2}$Te${}_{4}$
Bulk	*a*	0.4230	0.4300	0.4430
	*c*	3.954	4.042	4.156
	Slab thickness	1.037	1.072	1.127
	b1	0.3029	0.3051	0.3070
	b2	0.2987	0.3085	0.3248
	b3	0.2958	0.3046	0.3210
Nanosheet	*a*	0.4210	0.4280	0.4410
	*c*	1.041	1.074	1.128
	b1	0.3018	0.3030	0.3048
	b2	0.2987	0.3082	0.3244
	b3	0.2959	0.3047	0.3203

**Table 2:** Bulk modulus *B* (GPa), bulk elastic constants C${}_{ij}$ (GPa), two-dimensional elastic constant $C_{2D}$ (N/m), effective mass $m^{*}$ ($m_{e}$) of electrons/holes, cohesive energy $E_{coh}$ (eV/at.) at 0 K, calculated with the WC–GGA functional. (Note: The column corresponding to the deformation potential constants has been deleted).

	*B*	$C_{11}$	$C_{12}$	$C_{13}$	$C_{14}$	$C_{33}$	$C_{44}$	$C_{2D}$	$m^{*}$	$E_{coh}$
PbBi${}_{2}$Te${}_{2}$S${}_{2}$	30.33	50.2	18.2	23.8	14.1	41.1	27.9	21.2	−0.023/0.073	−3.22
PbBi${}_{2}$Te${}_{2}$Se${}_{2}$	28.75	49.5	14.5	22.7	13.5	40.2	27.0	21.2	−0.024/0.073	−3.09
PbBi${}_{2}$Te${}_{4}$	26.05	44.5	13.0	20.7	13.1	36.4	35.6	19.6	−0.028/0.085	−2.93

**Table 3:** Calculated lattice parameters a (nm), thickness c (nm), and relative energy $E_{re}=E_{\mathrm{strained}}-E_{\mathrm{unstrained}}$ ($10^{-2}$ eV) of PbBi${}_{2}$Te${}_{2}$S${}_{2}$, PbBi${}_{2}$Te${}_{2}$Se${}_{2}$ and PbBi${}_{2}$Te${}_{4}$ monolayers under strains η.

Strains	PbBi${}_{2}$Te${}_{2}$S${}_{2}$			PbBi${}_{2}$Te${}_{2}$Se${}_{2}$			PbBi${}_{2}$Te${}_{4}$		
	a	c	$E_{\mathrm{re}}$	a	c	$E_{\mathrm{re}}$	a	c	$E_{\mathrm{re}}$
−3%	4.09	10.76	8.28	4.15	11.10	10.41	4.27	11.65	9.93
−2%	4.13	10.64	3.19	4.20	10.98	4.99	4.32	11.53	4.90
−1%	4.17	10.52	0.5	4.24	10.86	1.84	4.36	11.40	1.92
0%	4.21	10.41	0	4.28	10.74	0	4.41	11.28	0
1%	4.26	10.29	0.7	4.33	10.62	1.72	4.45	11.16	1.70
2%	4.30	10.17	3.68	4.37	10.50	4.41	4.49	11.04	4.31
3%	4.34	10.05	8.74	4.41	10.38	8.90	4.54	10.91	8.52

## Data Availability

See Supplementary data on MDPI website.

## Supplementary Materials

The following are available online at https://www.mdpi.com/article/10.3390/nano11112979/s1, Figure S1: Primitive (a) and conventional (b) cell of bulk PbBi${}_{2}$Te${}_{4}$. Figure S2: Calculated electronic band structures of PbBi${}_{2}$Te${}_{4}$ with WC-GGA (blue lines) and HSE06 hybrid (red lines) functionals. Figure S3: Calculated partial DOS of PbBi${}_{2}$Te${}_{2}$S${}_{2}$, PbBi${}_{2}$Te${}_{2}$Se${}_{2}$ and PbBi${}_{2}$Te${}_{4}$ with WC-GGA functionals. Figure S4: (a,b,c) Volume dependence of the free energy from 0 K to 900 K with a temperature step of 100 K, and (d,e,f) temperature dependence of the crystal volume. PbBi${}_{2}$Te${}_{2}$S${}_{2}$ (a,d), PbBi${}_{2}$Te${}_{2}$Se${}_{2}$ (b,e) and PbBi${}_{2}$Te${}_{4}$ (c,f). Figure S5: Calculated Gibbs energy as a function of temperature for PbBi${}_{2}$Te${}_{2}$S${}_{2}$ (a), PbBi${}_{2}$Te${}_{2}$Se${}_{2}$ (b) and PbBi${}_{2}$Te${}_{4}$ (c). Figure S6: Group velocities (left panel) and phonon lifetimes (right panel) in the xx direction at 300 K for PbBi${}_{2}$Te${}_{2}$S${}_{2}$ (top row), PbBi${}_{2}$Te${}_{2}$Se${}_{2}$ (middle row), and PbBi${}_{2}$Te${}_{4}$ (bottom row). Contribution from phonon mode ZA (blue circles), TA (orange circles), LA (purple circles), optical (olive circles) branches and averaged value over a small frequency window of 0.04 THz (red lines). Figure S7: τ-scaled power factor of PbBi${}_{2}$Te${}_{2}$S${}_{2}$ monolayer versus p-type doping level for various compressive and tensile strains in a-axis direction at 500 K. Figure S8: Phonon spectrum curves of PbBi${}_{2}$Te${}_{2}$Se${}_{2}$ monolayer under strains. Figure S9: Phonon spectrum curves of PbBi${}_{2}$Te${}_{4}$ monolayer under strains. Table S1: Born effective charges $Z^{*}$ (e) and dielectric constants $\epsilon_{\infty }$ in the in-layer and cross-layer directions [xx, zz] of PbBi${}_{2}$Te${}_{2}$S${}_{2}$, PbBi${}_{2}$Te${}_{2}$Se${}_{2}$ and PbBi${}_{2}$Te${}_{4}$ calculated with the LDA (Ceperley-Alder) functional.

## References

[1] Lu X., Zhang Q., Liao J., Chen H., Fan Y., Xing J., Gu S., Huang J., Ma J., Wang J. (2020). High-Efficiency Thermoelectric Power Generation Enabled by Homogeneous Incorporation of MXene in (Bi,Sb)_2_Te_3_ Matrix. Adv. Energy Mater..

[2] Liu W., Jie Q., Kim H.S., Ren Z. (2015). Current Progress and Future Challenges in Thermoelectric Power Generation: From Materials to Devices. Acta Mater..

[3] Chowdhury I., Prasher R., Lofgreen K., Chrysler G., Narasimhan S., Mahajan R., Koester D., Alley R., Venkatasubramanian R. (2009). On-Chip Cooling by Superlattice-Based Thin-Film Thermoelectrics. Nat. Nanotechnol..

[4] Hubbard W.A., Mecklenburg M., Lodico J.J., Chen Y., Ling X.Y., Patil R., Kessel W.A., Flatt G.J.K., Chan H.L., Vareskic B. (2020). Electron-Transparent Thermoelectric Coolers Demonstrated with Nanoparticle and Condensation Thermometry. ACS Nano.

[5] Vining C.B. (2009). An Inconvenient Truth about Thermoelectrics. Nat. Mater..

[6] Zhu Q., Song S., Zhu H., Ren Z. (2019). Realizing High Conversion Efficiency of Mg_3_Sb_2_-Based Thermoelectric Materials. J. Power Sour..

[7] Balout H., Boulet P., Record M.C. (2017). Strain-Induced Electronic Band Convergence: Effect on the Seebeck Coefficient of Mg_2_Si for Thermoelectric Applications. J. Mol. Model..

[8] Diznab M.R., Maleki I., Vaez Allaei S.M., Xia Y., Naghavi S.S. (2019). Achieving an Ultrahigh Power Factor in Sb_2_Te_2_Se Monolayers via Valence Band Convergence. ACS Appl. Mater. Interfaces.

[9] Thébaud S., Adessi C., Pailhès S., Bouzerar G. (2017). Boosting the Power Factor with Resonant States: A Model Study. Phys. Rev. B.

[10] Heremans J.P., Wiendlocha B., Chamoire A.M. (2012). Resonant Levels in Bulk Thermoelectric Semiconductors. Energy Environ. Sci..

[11] Zhang Q., Wang H., Zhang Q., Liu W., Yu B., Wang H., Wang D., Ni G., Chen G., Ren Z. (2012). Effect of Silicon and Sodium on Thermoelectric Properties of Thallium-Doped Lead Telluride-Based Materials. Nano Lett..

[12] Zhang G., Kirk B., Jauregui L.A., Yang H., Xu X., Chen Y.P., Wu Y. (2012). Rational Synthesis of Ultrathin N-Type Bi_2_Te_3_ Nanowires with Enhanced Thermoelectric Properties. Nano Lett..

[13] Wu H.J., Zhao L.D., Zheng F.S., Wu D., Pei Y.L., Tong X., Kanatzidis M.G., He J.Q. (2014). Broad Temperature Plateau for Thermoelectric Figure of Merit ZT>2 in Phase-Separated PbTe_0.7_S_0.3_. Nat. Commun..

[14] Kim H.S., Kim S.I., Lee K.H., Kim S.W., Snyder G.J. (2017). Phonon Scattering by Dislocations at Grain Boundaries in Polycrystalline Bi_0.5_Sb_1.5_Te_3_. Phys. Status Solidi (b).

[15] Wu H., Lu X., Wang G., Peng K., Chi H., Zhang B., Chen Y., Li C., Yan Y., Guo L. (2018). Sodium-Doped Tin Sulfide Single Crystal: A Nontoxic Earth-Abundant Material with High Thermoelectric Performance. Adv. Energy Mater..

[16] Lu Z., Wu Y., Xu Y., Ma C., Chen Y., Xu K., Zhang H., Zhu H., Fang Z. (2019). Ultrahigh Electron Mobility Induced by Strain Engineering in Direct Semiconductor Monolayer Bi_2_TeSe_2_. Nanoscale.

[17] Mishra P., Singh D., Sonvane Y., Ahuja R. (2020). Two-Dimensional Boron Monochalcogenide Monolayer for Thermoelectric Material. Sustain. Energy Fuels.

[18] Sun Y., Cheng H., Gao S., Liu Q., Sun Z., Xiao C., Wu C., Wei S., Xie Y. (2012). Atomically Thick Bismuth Selenide Freestanding Single Layers Achieving Enhanced Thermoelectric Energy Harvesting. J. Am. Chem. Soc..

[19] Mounet N., Gibertini M., Schwaller P., Campi D., Merkys A., Marrazzo A., Sohier T., Castelli I.E., Cepellotti A., Pizzi G. (2018). Two-Dimensional Materials from High-Throughput Computational Exfoliation of Experimentally Known Compounds. Nat. Nanotechnol..

[20] Ambrosi A., Pumera M. (2018). Exfoliation of Layered Materials Using Electrochemistry. Chem. Soc. Rev..

[21] Chatterjee A., Biswas K. (2015). Solution-Based Synthesis of Layered Intergrowth Compounds of the Homologous Pb_m_Bi_2n_Te_3n+m_ Series as Nanosheets. Angew. Chem. Int. Ed..

[22] Park K., Heremans J.J., Scarola V.W., Minic D. (2010). Robustness of Topologically Protected Surface States in Layering of Bi_2_Te_3_ Thin Films. Phys. Rev. Lett..

[23] Shvets I.A., Klimovskikh I.I., Aliev Z.S., Babanly M.B., Sánchez-Barriga J., Krivenkov M., Shikin A.M., Chulkov E.V. (2017). Impact of Stoichiometry and Disorder on the Electronic Structure of the PbBi_2_Te_4−x_Se_x_ Topological Insulator. Phys. Rev. B.

[24] Peng R., Ma Y., Wang H., Huang B., Dai Y. (2020). Stacking-Dependent Topological Phase in Bilayer MBi_2_Te_4_ (M = Ge, Sn, Pb). Phys. Rev. B.

[25] Hung N.T., Nugraha A.R., Saito R. (2019). Designing High-Performance Thermoelectrics in Two-Dimensional Tetradymites. Nano Energy.

[26] Sharma S., Schwingenschlögl U. (2016). Thermoelectric Response in Single Quintuple Layer Bi_2_Te_3_. ACS Energy Lett..

[27] Blaha P., Schwarz K., Tran F., Laskowski R., Madsen G.K.H., Marks L.D. (2020). WIEN2k: An APW+lo Program for Calculating the Properties of Solids. J. Chem. Phys..

[28] Madsen G.K., Carrete J., Verstraete M.J. (2018). BoltzTraP2, a Program for Interpolating Band Structures and Calculating Semi-Classical Transport Coefficients. Comput. Phys. Commun..

[29] Giannozzi P., Baroni S., Bonini N., Calandra M., Car R., Cavazzoni C., Ceresoli D., Chiarotti G.L., Cococcioni M., Dabo I. (2009). Quantum ESPRESSO: A Modular and Open-Source Software Project for Quantum Simulations of Materials. J. Phys. Condens. Matter.

[30] Togo A., Chaput L., Tanaka I. (2015). Distribution of Phonon Lifetime in Brillouin Zone. Phys. Rev. B.

[31] Chaput L. (2013). Direct Solution to the Linearized Phonon Boltzmann Equation. Phys. Rev. Lett..

[32] Ma W., Record M.C., Tian J., Boulet P. (2021). Strain Effects on the Electronic and Thermoelectric Properties of n(PbTe)-m(Bi_2_Te_3_) System Compounds. Materials.

[33] Wu Z., Cohen R.E. (2006). A More Accurate Generalized Gradient Approximation for Solids. Phys. Rev. B.

[34] Shelimova L.E., Karpinskii O.G., Konstantinov P.P., Avilov E.S., Kretova M.A., Zemskov V.S. (2004). Crystal Structures and Thermoelectric Properties of Layered Compounds in the ATe–Bi_2_Te_3_ (A = Ge, Sn, Pb) Systems. Inorg. Mater..

[35] Heyd J., Peralta J.E., Scuseria G.E., Martin R.L. (2005). Energy Band Gaps and Lattice Parameters Evaluated with the Heyd-Scuseria-Ernzerhof Screened Hybrid Functional. J. Chem. Phys..

[36] Park S., Ryu B. (2016). Hybrid-Density Functional Theory Study on Band Structures of Tetradymite-Bi_2_Te_3_, Sb_2_Te_3_, Bi_2_Se_3_, and Sb_2_Se_3_ Thermoelectric Materials. J. Korean Phys. Soc..

[37] Mouhat F., Coudert F.X. (2014). Necessary and Sufficient Elastic Stability Conditions in Various Crystal Systems. Phys. Rev. B.

[38] Campi D., Paulatto L., Fugallo G., Mauri F., Bernasconi M. (2017). First-Principles Calculation of Lattice Thermal Conductivity in Crystalline Phase Change Materials: GeTe, Sb_2_Te_3_, and Ge_2_Sb_2_Te_5_. Phys. Rev. B.

[39] Ceperley D.M., Alder B.J. (1980). Ground State of the Electron Gas by a Stochastic Method. Phys. Rev. Lett..

[40] Hellman O., Broido D.A. (2014). Phonon Thermal Transport in Bi_2_Te_3_ from First Principles. Phys. Rev. B.

[41] Zhang Y., Ke X., Chen C., Yang J., Kent P.R.C. (2009). Thermodynamic Properties of PbTe, PbSe, and PbS: A First-Principles Study. Phys. Rev. B.

[42] Ma W., Record M.C., Tian J., Boulet P. (2021). Influence of the Stacking Sequence on Layered-Chalcogenides Properties: First Principle Investigation of Pb_2_Bi_2_Te_5_. Phys. Chem. Chem. Phys..

[43] King-Smith R., Vanderbilt D. (1993). Theory of Polarization of Crystalline Solids. Phys. Rev. B.

[44] Slack G.A. (1973). Nonmetallic crystals with high thermal conductivity. J. Phys. Chem. Solids.

[45] Shalímova K. (1982). Fisica de los Semiconductors.

[46] Morelli D.T., Heremans J.P. (2002). Thermal Conductivity of Germanium, Silicon, and Carbon Nitrides. Appl. Phys. Lett..

