# Supplementary material for: First-Principle Investigations on the Electronic and Transport Properties of PbBi2Te2X2 (X = S/Se/Te) Monolayers
Source: Nanomaterials (Basel). 2021 Nov 5;11(11):2979. doi: 10.3390/nano11112979 (PMC8624905; doi:10.3390/nano11112979)
Supplement: Supplementary file 1 [file nanomaterials-11-02979-s001.zip › nanomaterials-1407143-supplementary.pdf]

nanomaterials

*Supplementary Materials*

# First-Principle Investigations on the Electronic and Transport Properties of $PbBi_2Te_2X_2$ (X=S/Se/Te) Monolayers

**Weiliang Ma[1,2], Jing Tian[1,2], Pascal Boulet[2] and Marie-Christine Record[1,*]**

[1]Aix-Marseille University, Faculty of Sciences, CNRS,IM2NP, 13013 Marseille, France; weiliang.ma@etu.univ-amu.fr (W. M.); jing.tian@etu.univ-amu.fr (J. T.)
[2]Aix-Marseille University, Faculty of Sciences, CNRS, MADIREL, 13013 Marseille, France; pascal.boulet@univ-amu.fr,
* Correspondence: Correspondence: m-c.record@univ-amu.fr

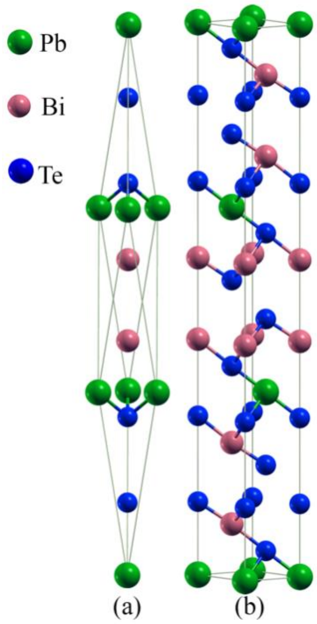


**Figure S1.** Primitive (**a**) and conventional (**b**) cell of bulk $PbBi_2Te_4$.

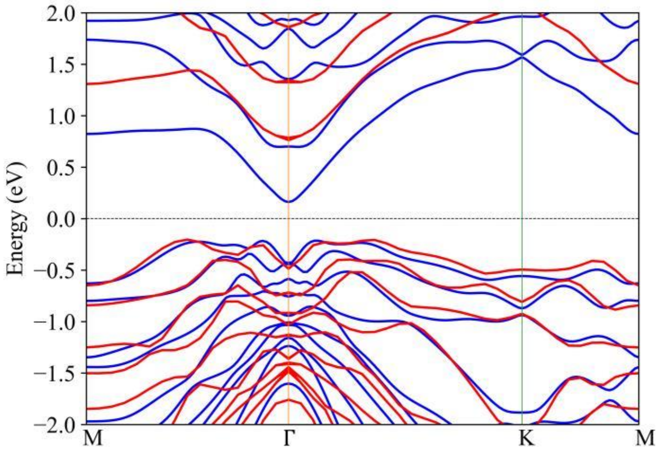


**Figure S2.** Calculated electronic band structures of $PbBi_2Te_4$ with the WC GGA (blue lines) and HSE06 hybrid (red lines) functionals.

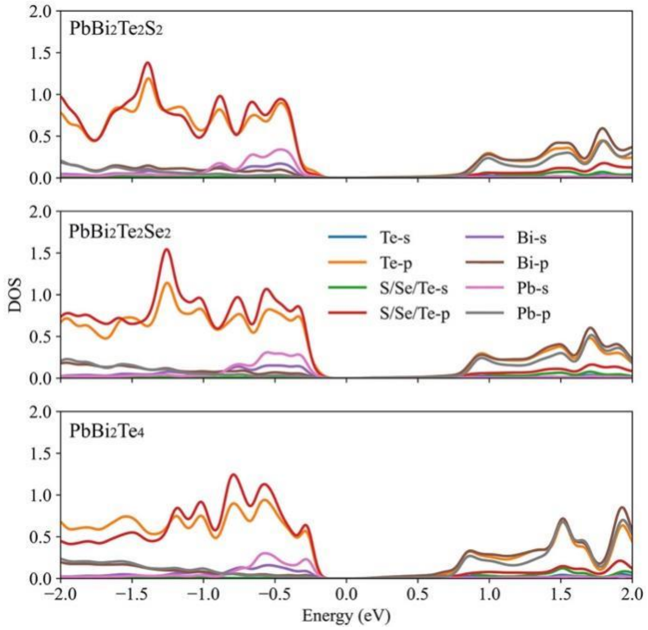


**Figure S3.** Calculated partial DOS of $PbBi_2Te_2S_2$, $PbBi_2Te_2Se_2$ and $PbBi_2Te_4$ with the WC functional.

**Table S1.** Born effective charges Z* (e) and dielectric constants $\varepsilon_\infty$ in the in-layer and cross-layer directions [*xx*, *zz*] of $PbBi_2Te_2S_2$, $PbBi_2Te_2Se_2$ and $PbBi_2Te_4$ calculated with the LDA (Ceperley-Alder) functional.

| | $Z^*_{Pb}$ | $Z^*_{Bi}$ | $Z^*_{Te}$ | $Z^*_{S/Se/Te}$ | $\varepsilon_\infty$ |
|---|---|---|---|---|---|
| $Pb_2Bi_2Te_2S_2$ | [4.88, 0.81] | [5.76, 0.59] | [-5.27, -0.80] | [-2.93, -0.21] | [12.73, 9.43] |
| $Pb_2Bi_2Te_2Se_2$ | [5.05, 0.85] | [6.17, 0.61] | [-5.25, -0.83] | [-3.45, -0.21] | [13.32,11.00] |
| $Pb_2Bi_2Te_4$ | [5.34, 0.98] | [7.08, 0.65] | [-5.28, -0.93] | [-4.48, -0.22] | [16.09,13.59] |

**Thermodynamic stability of the $PbBi_2Te_2S_2$, $PbBi_2Te_2Se_2$ and $PbBi_2Te_4$ compounds:**

To evaluate the thermodynamic stability of our compounds, we have calculated the Gibbs energy using the quasi-harmonic approximation (QHA) approach [1] in which the phonons are harmonic, but they depend on volume. The Gibbs free energy G(T,P) is given as:

$$G(T, P) = F(T, V) + PV$$

where $F(T, V)$ is the Helmholtz free energy at temperature T and volume V, and P is the pressure. The free energy of a crystalline material $F(T, V)$ can be expressed as follows [2]:

$$F(T, V) = E(V)_{0K} + F_{vib}(T) + F_{el}(T, V)$$

where $E(V)_{0K}$ is the energy at 0K, $F_{vib}(T, V)$ is the vibrational free energy and $F_{el}(T, V)$ is the electronic free energy. The vibrational free energy reads [2]

$$F_{vib}(T) = \frac{1}{2}\sum_{k,\nu} \hbar\omega(k, \nu) + k_B T \sum_{k,\nu} \ln\left(1 - \exp\frac{\hbar\omega(k, \nu)}{k_B T}\right)$$

where $\omega(k, \nu)$ is the phonon frequency of the $k^{th}$ band and $\nu^{th}$ vector. In the following, the electronic part is neglected as this contribution is usually small.

The Gibbs energy $G(T, P)$ has been calculated at constant pressure and temperature by selecting the minimum value for each volume change (red line in Fig. S4). Then, the Gibbs energy is plotted as a function of temperature as shown in Fig. S5. Negative G is found for the investigated monolayers, suggesting that they are all stable.

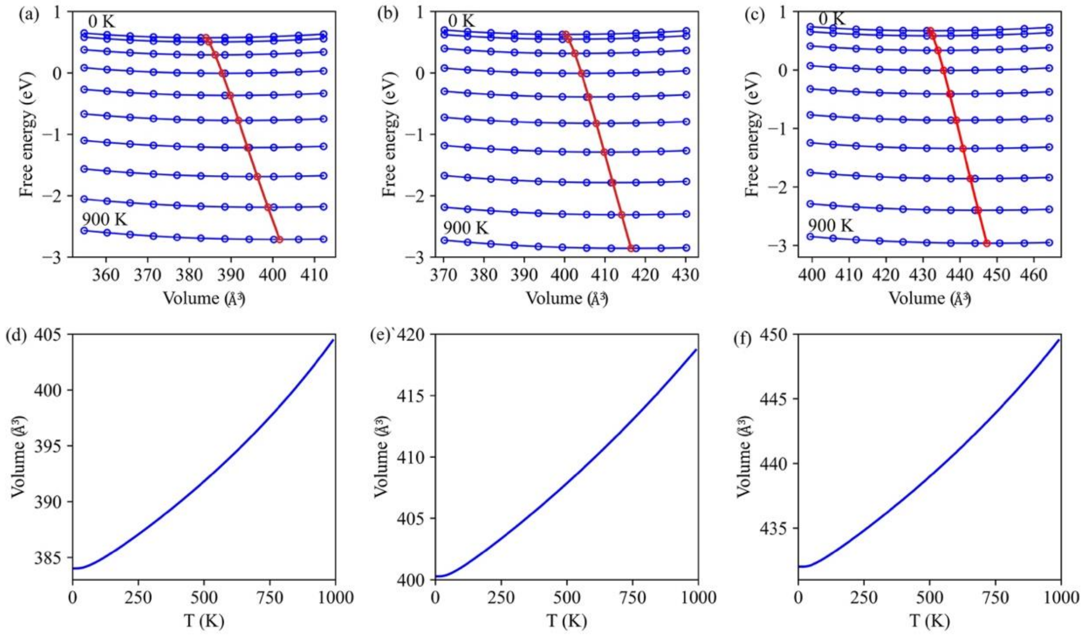


**Figure S4.** (**a,b,c**) Volume dependence of the free energy from 0K to 900K with a temperature step of 100K, and (**d,e,f**) temperature dependence of the crystal volume. $PbBi_2Te_2S_2$ (**a, d**), $PbBi_2Te_2Se_2$ (**b, e**) and $PbBi_2Te_4$ (**c, f**).

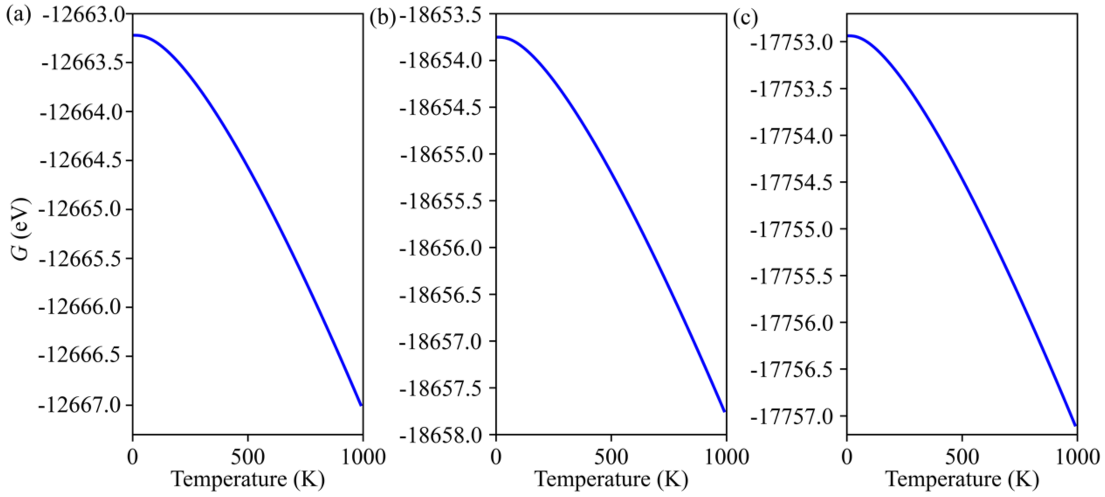


**Figure S5.** Calculated Gibbs energy as a function of temperature for $PbBi_2Te_2S_2$ (**a**), $PbBi_2Te_2Se_2$ (**b**) and $PbBi_2Te_4$ (**c**).

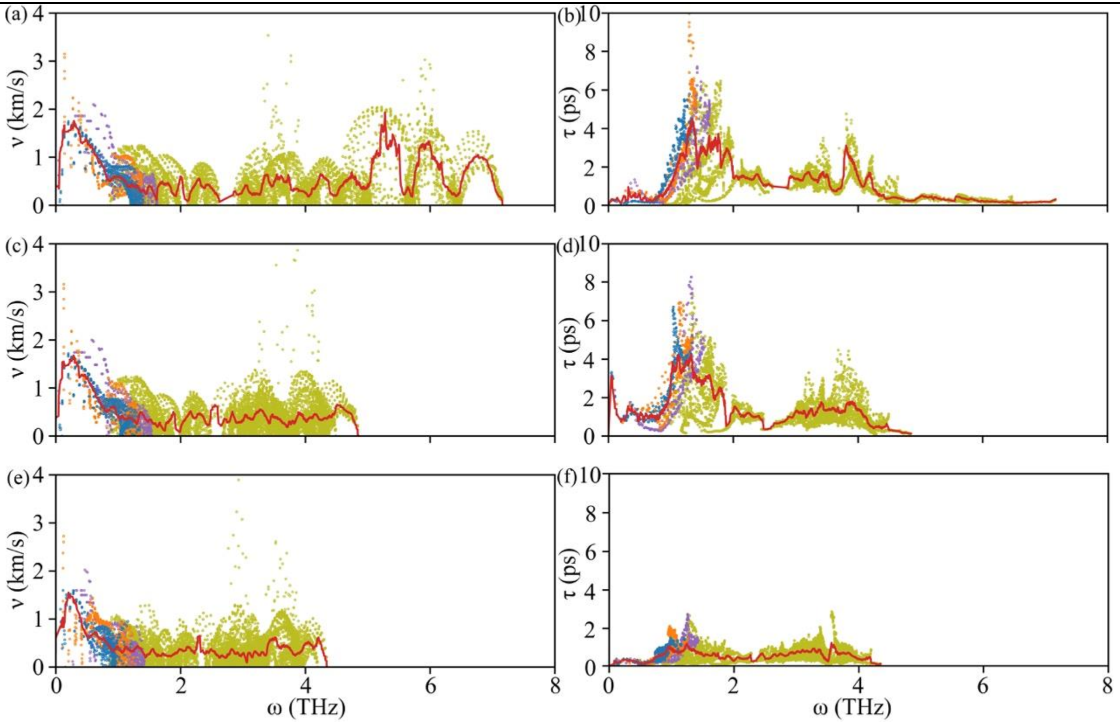


**Figure S6.** Group velocities (left panel) and phonon lifetimes (right panel) in the xx direction at 300K for $PbBi_2Te_2S_2$ (top row), $PbBi_2Te_2Se_2$ (middle row), and $PbBi_2Te_4$ (bottom row). Contribution from phonon mode ZA (blue circles), TA (orange circles), LA (purple circles), optical (olive circles) branches and averaged value over a small frequency window of 0.04 THz (red lines).

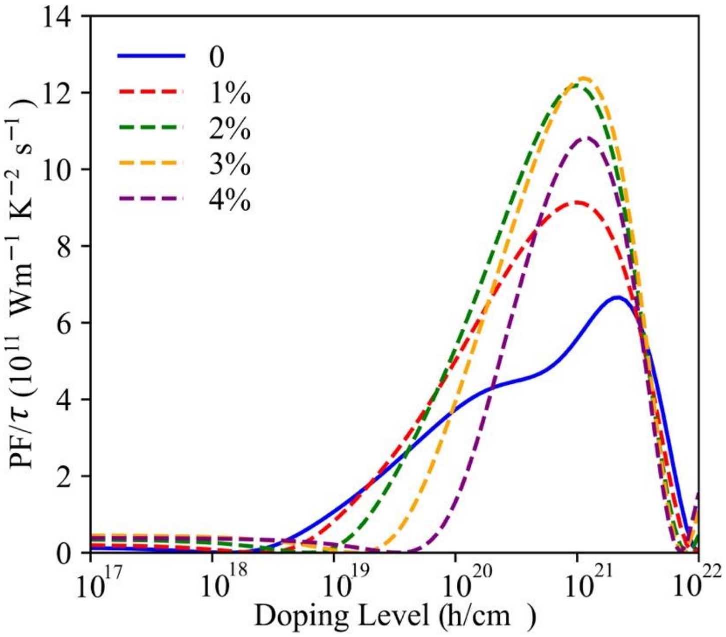


**Figure S7.** τ-scaled power factor of $PbBi_2Te_2S_2$ monolayer versus p-type doping level for various compressive and tensile strains in a-axis direction at 500K.

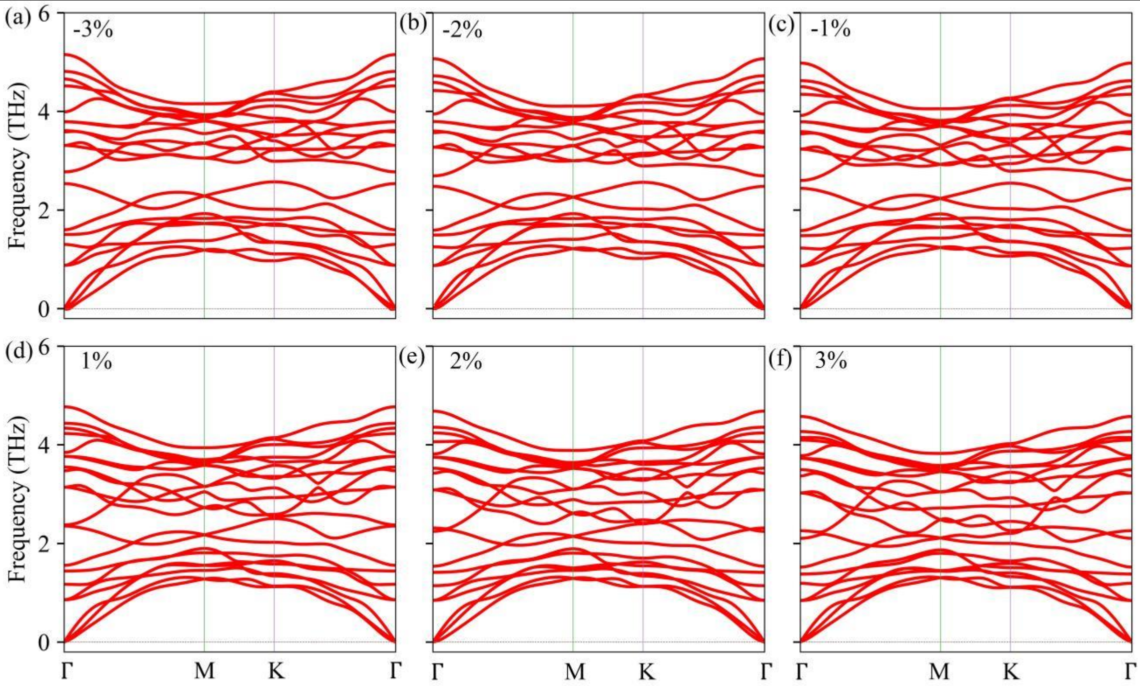


**Figure S8.** Phonon spectrum curves of $PbBi_2Te_2Se_2$ monolayer under strains.

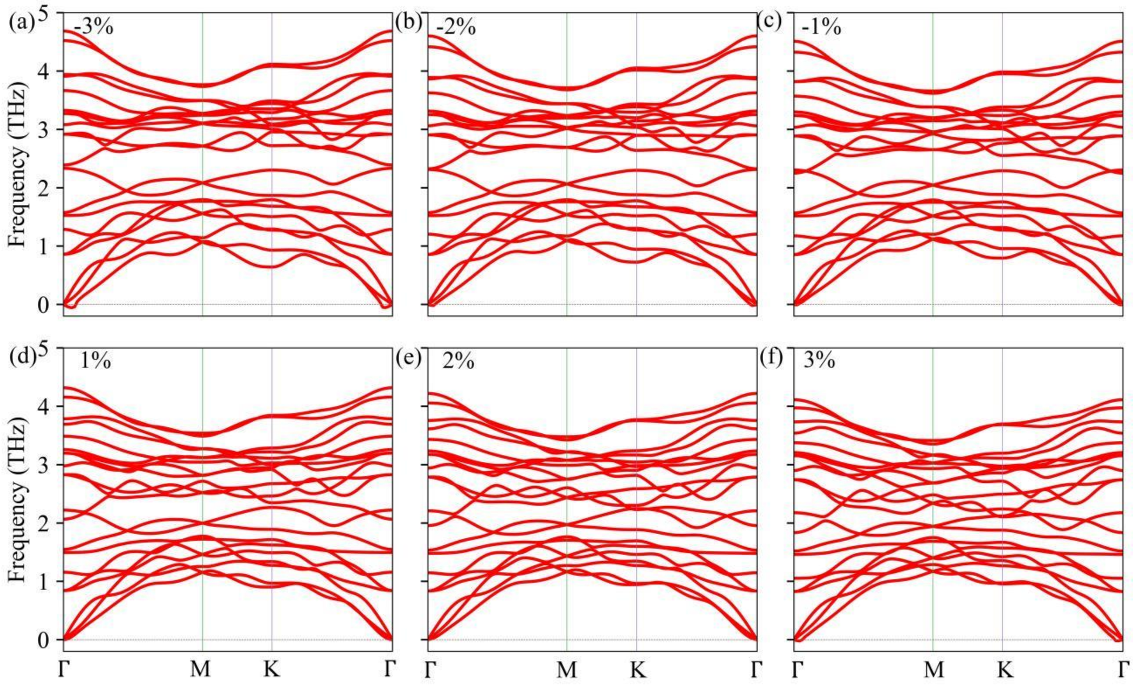


**Figure S9.** Phonon spectrum curves of $PbBi_2Te_4$ monolayer under strains.
